# Discussing poverty within primary care consultations: implications for mental health support

**DOI:** 10.3399/BJGPO.2024.0249

**Published:** 2025-05-07

**Authors:** Felicity Thomas, Katrina Wyatt, Kathryn Berzins, Ilse Lee, Jane Horrell, Alison McLoughlin

**Affiliations:** 1 Department for Health and Community Sciences, University of Exeter, Exeter, UK; 2 Health Technology Assessment Unit, Applied Health Research Hub, University of Central Lancashire, Preston, UK; 3 UCLPartners, London, UK; 4 Faculty of Health: Medicine, Dentistry and Human Sciences, University of Plymouth, Plymouth, UK; 5 Royal Blackburn Teaching Hospital, Blackburn, UK

**Keywords:** Mental health, Poverty, Difficult conversations, Socioeconomic factors, Primary health care

## Abstract

**Background:**

Poverty can have significant impacts on health and wellbeing. However, asking patients about their broader socioeconomic circumstances is not routine within primary care consultations.

**Aim:**

To understand healthcare professionals’ experiences of communicating with patients about their socioeconomic circumstances and how a bespoke training programme supported these conversations in routine consultations.

**Design & setting:**

Healthcare professionals from 30 GP practices across England received training to improve understanding and communication with patients about the ways that poverty impacted their mental health.

**Method:**

Semi-structured interviews were undertaken with 49 GPs and allied health professionals to understand barriers and enablers to communication around poverty and the impact of the training on their consultation practice.

**Results:**

Health professionals often lacked confidence in discussing socioeconomic issues and welcomed information on how to do this sensitively. Asking questions about poverty-related stresses was felt to lead to better understanding of the causes of mental distress, avoidance of problematic assumptions and inappropriate antidepressant prescribing, and to enable more coordinated and appropriate support from practice teams.

**Conclusion:**

Asking patients about their socioeconomic circumstances can facilitate provision of appropriate support.

## How this work fits in

Although poverty is associated with poor mental health, our work found that it was not routinely discussed with patients within GP practice settings. Research on having difficult conversations with patients has found that practitioners do not always know how to broach issues that might seem sensitive or know how to respond to them effectively. We worked with GPs and partners within low-income communities to develop training to help practitioners feel confident in asking patients about their broader socioeconomic circumstances. Participants reported increased understanding of the causes of mental distress, provision of more appropriate treatment and support, and better practice team coordination.

## Introduction

The past decade has witnessed deepening levels of poverty in the UK alongside intense resource pressures on primary care. Socioeconomic factors are reported as being the largest determinant of health and wellbeing,^1^ with strong associations between poverty and poor mental and physical health.^2^ Yet while these issues have a significant impact on workload within primary care, socioeconomic circumstances are not routinely discussed within consultations. This paper draws on research examining ‘difficult conversations’^3^ and the challenges associated with discussing ‘emotional concerns’ relating to social stressors within primary care^4^ to explore how training helped GP practice teams to feel more confident in asking about and responding to patients experiencing poverty-related mental distress.

Engaging with emotional concerns has a range of important ramifications for diagnosis and treatment – providing clues to underlying psychological and physical issues, and potentially enabling discussion which may change patient beliefs about their ill health and/or lead to greater treatment acceptance and adherence, as well as helping build a therapeutic alliance and improving patient satisfaction.^5^ Similarly, displaying empathetic concern is reported to contribute to consultation quality for patients with low socioeconomic status,^6^ with the degree of physician empathy being associated with patient enablement post-consultation.^7^

Despite these benefits, research has shown wide variability in how patients raise, and GPs enquire about and engage with, emotional concerns. A systematic review found that patients from lower socioeconomic backgrounds receive a more directive and less participatory consulting style than others.^8^ This coheres with research on patient experiences of mental health consultations where those from low-income backgrounds commonly report reluctance to disclose socioeconomic problems as a result of stigma, feeling that their concerns will be dismissed, and that ensuing treatment plans are likely to be inappropriate to their needs.^9,10^

Self-reported barriers to GPs discussing emotional concerns with patients include a lack of clarity around the extent and focus of their roles and responsibilities,^11,12^ feeling under-skilled,^13^ and uncertainty over when and how to elicit and respond to these conversations.^3,4^ The prioritisation of competing demands, concerns around consultation time and resource pressures are also barriers, despite evidence suggesting that explicit acknowledgement of emotional concerns is associated with reduced consultation length^14^ and can decrease lengthy follow-up consultations.^6^

### The DeStress-II training programme

The DeStress-II training resource was developed in collaboration with partners within low-income communities and GPs to help primary care practitioners deliver more effective consultations for patients presenting with poverty-related mental distress (https://destressproject.org.uk/supporting-effective-consultations/). An initial version of the training was piloted (in-person and online depending on COVID-19 restrictions at the time) with 500 primary care practitioners (387 GPs and 113 allied health professionals) in 53 practices across three English regions selected to include diverse poverty-affected populations: the south west (rural, coastal and post-industrial areas of Somerset, Devon, Cornwall); north Thames (inner-city London; urban/semi-urban south Essex) and the north west coast (inner city Liverpool, post-industrial and coastal Lancashire). Training was delivered by teams comprising a GP, a community partner, and a researcher. Feedback from this training (elicited in reflective discussion at the end of the training and in follow-up interviews with 22 health professionals) was then used to better understand the barriers and enablers that health practitioners faced in supporting patients experiencing poverty-related mental distress and this was drawn on to further refine the training.

One area that emerged repeatedly in this feedback was the (un)willingness of and challenges faced by many health professionals in eliciting discussion around a patient’s broader life circumstances. The DeStress-II project community partners felt that this could have problematic implications for patients – both in terms of their experience within the consultation and the appropriateness of the response and/or treatment received. Furthermore, earlier feedback from patients (*n *= 107) who had received a consultation influenced by the pilot phase of the training was particularly positive when patients felt they had been able to talk about their wider concerns and feel listened to (as discussed by Thomas *et al* ^6^). A focus on acknowledging and engaging with a patient’s broader life circumstances and the particular challenges of poverty therefore became a central theme in the training resource developed.

## Method

An online training resource that could be facilitated by a member of the practice team was identified as being the most effective way of reaching a large volume of primary care practitioners. Using feedback from initial piloting, we co-developed an online resource comprising information slides, film clips of professional and patient experience, consultation role play, scripts for use within consultations and questions for reflective group discussion. Topics covered included i) information on the links between poverty and mental health; ii) reflective team-based discussion on current consultation and treatment practice; iii) scripts and prompts to help develop engagement, shared biopsychosocial understanding, and open up discussion to acknowledge, validate and show empathy for patient experience and circumstance (for a more detailed discussion on the training development, delivery and outcomes see^6^). Practices nominated a staff member (in most cases a GP) to facilitate the training, and they were provided with a short instruction manual to support this role.

Over 150 health-care professionals from 30 GP practices across England participated in the (RCGP-accredited) DeStress-II online training. Table 1 details practice staff attending the training.

**Table 1.** Primary care staff receiving DeStress-II training

Staff role	Participants, *n*
GP	72
GP registrar	17
Nurse	25
Pharmacist	9
Social prescriber	8
Physician associate	2
Mental health practitioner	5
Health care assistant	3
Care coordinator	3
Advanced care practitioner	4
Other for example practice manager/admin	10
Total	158

a We were unable to confirm participant numbers and roles in one practice.

Practices where the online training had taken place were invited to nominate up to two staff to participate in an interview to better understand how they perceived the training, any barriers and enablers to implementing the messages conveyed and any early impact on practice. A total of 49 healthcare professionals (from 29 practices) participated in interviews (online or via telephone) about their own perceptions and experiences, and the perspectives of others from their practice. The opportunity to participate in training and an interview was available to all practice staff undertaking patient consultations, including GPs, pharmacists, nurses, social prescribers, and healthcare assistants, as well as administrative staff (see Table 2).

**Table 2.** Primary care staff interviewed

Staff role	Participants, *n*
GP	26
GP registrar	1
Nurse	3
Pharmacist	2
Social prescriber	5
Physician associate	0
Mental health practitioner	2
Health care assistant	2
Care coordinator	2
Advanced care practitioner	2
Other for example practice manager/admin	4
Total	49

### Analysis

Reflexive thematic analysis^15^ was used to develop, analyse, and interpret patterns across the dataset. While a more inductive approach was used to identify the overall standout themes,^6^ our analysis was also influenced by earlier feedback from health professionals and community partners relating to discussions around life circumstances and poverty within consultations. This paper focuses on the more deductive analysis of data generated around health professionals’ pre-and post-training views and experiences of talking about a patient’s broader life circumstances and, in particular, how poverty may impact their mental wellbeing. A recursive six-step analytical approach was taken; first, ensuring familiarity with the data as it related to the discussion of life circumstances and poverty within consultations and reflecting on the broader context and constraints within which primary care professionals are working; second, generating initial codes which helped to organise and make sense of the data; third, searching for themes through examining coded data to trace repetition, as well as distinct features within and across participant data and how these related to questions around professional and (perceived) patient experience (including any cases that contradicted or challenged other findings), barriers and enablers to discussing poverty-related issues and implementing learning around this, and any early impact on practice; fourth, reviewing initial themes, making adjustments and clarifications to nuance analysis and ensure ongoing connection with the dataset. The fifth and sixth phases involved defining and naming themes, and confirming and contextualising findings. Initial coding was undertaken by the main author (FT) and shared with the other authors to discuss and reflect on assumptions made, and to identify any overlooked issues or themes. Participants have been anonymised in the reporting of findings.

## Results

Four core themes were identified in the data as they related to discussion of poverty-related issues: the need for health professionals to feel skilled and confident to ask patients questions about their socioeconomic circumstances; the importance of avoiding assumptions; reflecting on what constitutes appropriate support for mental distress underpinned by poverty; and the potential for more effective practice team working.

### Asking questions about socioeconomic circumstances

Research has shown that many patients from low-income backgrounds feel uncomfortable and disempowered within clinical encounters and are less likely than wealthier patients to voice their concerns, particularly in relation to stigmatised issues like poverty and mental health.^10,16^ Health professionals interviewed felt that it was uncommon for patients to share their circumstances without explicit prompting:

*‘It’s very rare that patients bring this [socio-economic circumstances] up other than for benefits forms.’* (GP10)

Research suggests that avoiding rather than normalising conversations around issues such as mental health and poverty can exacerbate stigma and result in ineffective or potentially harmful treatment responses.^16^ However, we found that the impacts of poverty on patient health and practice workload were not regularly discussed within practice teams. A minority of health professionals, in particular GPs, felt that asking about these issues was not within their remit and might trigger complex issues which they lacked the skills or resources to properly address. However, other interviewees reported lack of confidence and fear of offending as the main barriers:

*‘There’s a perception that maybe they* [patient] *don’t want to be asked and don’t want someone prying around too much in the personal, deeper personal information. But actually, I don’t think that’s true. I think people do want to be asked and I just think* [health professionals] *think they can’t ask*.’ (GP20)

Asking patients sensitive questions around suicide and substance use is mandated by guidance recommending risk assessment of patients with emotional concerns. Yet as one GP emphasised, really understanding the issues patients faced required consideration of a broader set of concerns, and active listening going beyond a ‘tick box’ approach:

*‘The social history is just like a tick box sometimes […] the medical students for example just ask about smoking, drinking — they think that’s a social history, but it’s not. There’s so much gambling, debt, the family structure and things. So, it’s about trying to go a bit deeper at the beginning*.’ (GP5)

**Figure 1.**
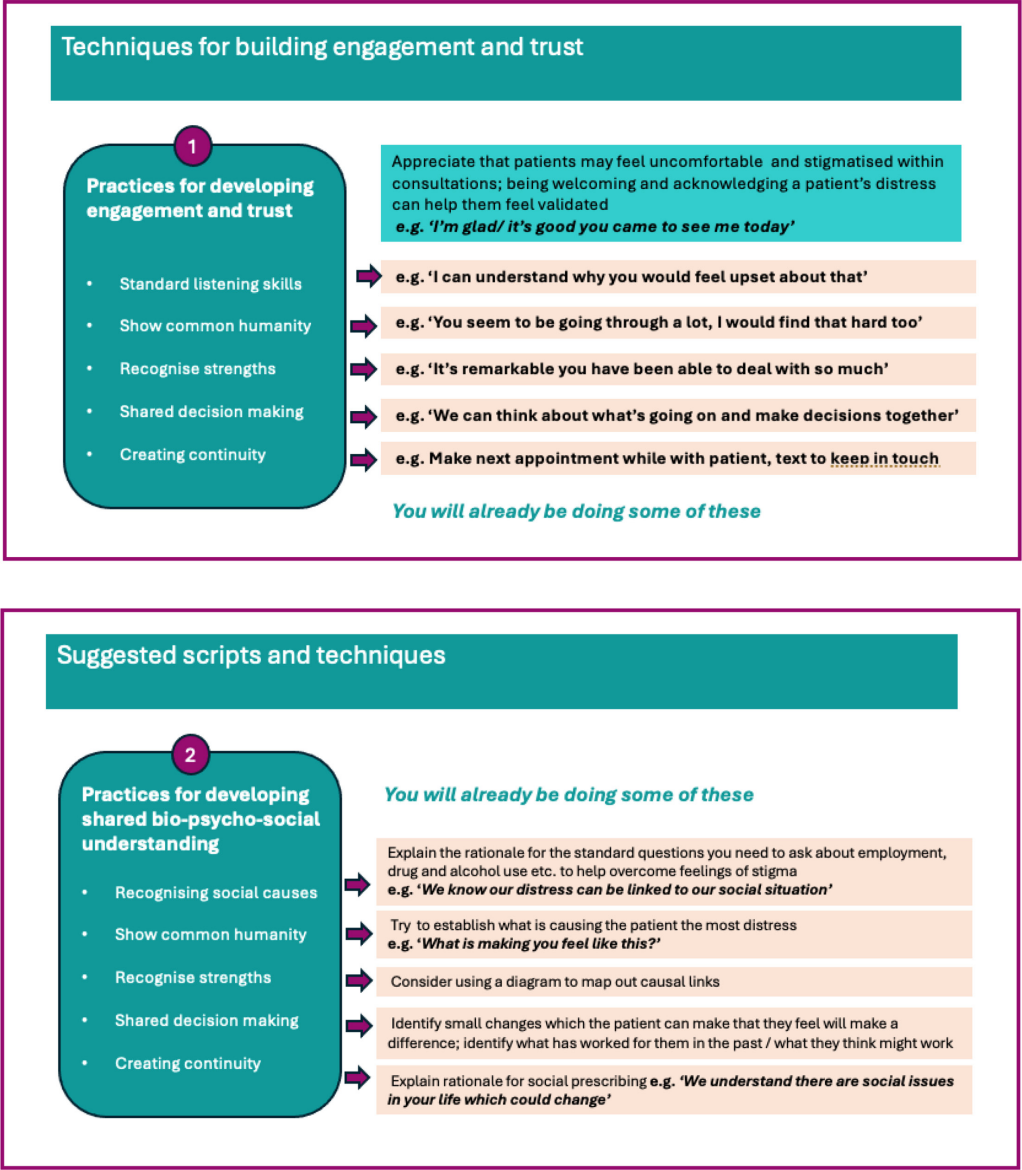
Examples of scripts and prompts within the training

The DeStress-II training contains scripts and prompts to acknowledge and help validate patient circumstances and open up space for non-judgmental discussion (see Figure 1).

GPs said the training had helped them to understand the broader challenges patients may be facing, and how they could use these scripts or prompts to engage in discussions which normalised patient responses to their mental distress as ‘what most people in your situation would feel’ rather than framing them as psychological problems necessarily requiring medical or therapy-based solutions:

*‘I thought the scripts given in the training about saying we are in this together, we can work together, I can support you — those scripts were quite useful.’* (GP02)

*‘I found* [training] *really thought provoking […] And it really made me think about what the patient’s journey actually is when they get to the surgery, all that comes before it, rather than just thinking about them being sat in front of you for those 10 minutes […]* [the training] *allowed me to think about and give myself permission to ask those questions.’* (GP20)

### Avoiding assumptions

Several GPs stressed how coming from very different backgrounds to their low-income patients meant that inaccurate assumptions could be made when questions relating to broader socioeconomic circumstances were not explicitly asked. GP10 stressed how important it was for health professionals to be continually aware of this difference, and the influence this might have on patient decision-making:

*‘Most doctors and healthcare professionals don’t come from a background where* [poverty] *is really experienced. So, I think we have to keep saying that our patients are on average quite different to us […] you know my washing machine broke and I didn’t have any money in the bank. That’s fine. Umpteen companies will offer me credit to buy a new one. But that isn’t true for a lot of our patients who have no personal or family or social safety […] and people will think differently about their choices*.’ (GP10)

The importance of avoiding assumptions was also highlighted by this GP as he explained how the current cost-of-living crisis meant financial problems could affect a wide range of patients:

*‘I think it’s easy to make assumptions in both ways. I’ve got patients who are on extremely low incomes, but who are really canny with their money and have always lived on very low incomes and are very effective in their use of that income to manage their life in the way that they want to. And other people, particularly where incomes have gone up and down, who I think often find it much more difficult*.’ (GP10)

### Rethinking appropriate support

Health professionals described how the DeStress-II training had helped them to better understand how mental health could be impacted by poverty-related stressors. This then prompted reconsideration of the types of support likely to be appropriate. One GP explained that he now felt able to recognise mental distress as being triggered by broader socioeconomic challenges rather than as something he would have previously defined as ‘clinical depression’ and ‘going to a single route of treatment’, through antidepressants. Questioning the appropriateness of prescribing medications for mental distress caused primarily by socioeconomic stressors was also raised:

*‘It seems a lot of it* [mental distress] *is to do with poverty and financial benefits being stopped, people being made redundant. A lot of people’s problems we probably shouldn’t be going anywhere near antidepressants for them. People come and say ‘I have been feeling really down, I’m feeling this’,* [we say] *‘here have a prescription’. Questions need to be asked.’* (Care coordinator)

Feeling confident and comfortable asking about socioeconomic circumstances early in the consultation process was also considered necessary to ensuring that potentially vulnerable patients received appropriate support and were prevented from entering a ‘revolving door’ of health concerns:

*‘It’s almost a safety net where if you don’t ask, they don’t open up and* [you] *leave a patient vulnerable. And that’s where I think, you know, the revolving door comes — they keep ringing for a physical or functional illness, which is really stemmed from some form of mental health, psychological trauma*.’ (Advanced nurse practitioner)

Asking patients about broader life circumstances and underlying stressors during initial consultations was also seen as vital when follow-up consultations were likely to be dominated by questions around medication experience and adherence:

*‘If patients have already gone down that route of medication, then the underlying issues can get left alone, or stay hidden because once the medication has started all the focus is, on the medication working or not working, less dose, put the dose up, change the medication*.’ (Clinical pharmacist)

Several health professionals described how their post-training confidence to ask questions had led to deeper insights about patients’ everyday challenges and had facilitated better signposting to support. Finance was an area that was especially challenging to raise, despite recognition that this would be central to people’s circumstances and mental wellbeing. Describing how he had previously ‘skirted around’ this issue, one GP explained how, through asking, he had been better able to understand the extent of the problems faced and provide more appropriate support for his patient:

*‘I’ve asked the question and been quite surprised they actually haven’t got enough money for food. I thought, oh God, I thought things were bad, but I didn’t think they were that bad […] but they would have never told you that unless you actually asked specifically. So just in terms of food vouchers and things that’s been quite surprising to me, how many people have been so grateful to have been asked about it and accepted it* [vouchers]*. And we think the people are ‘gonna feel stigmatised’, but actually, you know, they’re very grateful for the offer if it’s made*.’ (GP05)

While in this example, the GP was able to offer practical support via vouchers, this was not, under current resource constraints, always felt to be feasible — indeed, as reported elsewhere,^17^ inability (perceived or actual) to offer practical responses to support patient needs was identified as a barrier to asking questions in the first place. However, a survey with low-income patients (*n* = 107) experiencing mental distress undertaken as part of the wider study found that empathic listening and ‘feeling heard’ were core factors contributing to positive consultation experiences, suggesting that practical support was not a prerequisite to good practice.^6^

GP18 showed how, by asking such questions, they gained valuable insight into the stressors exacerbating their patient’s poor mental health, which led to a much broader discussion than would normally have been pursued:

*‘I did a consultation with someone I hadn’t seen before […] I was aware that I very much delved into his circumstances more than I perhaps would have done before the training. So now I’m aware that he’s suddenly got two children living with him who were living with their mum and this breakdown in relationship happened and she was in trouble with police and social care […].’* (GP18)

In this case, GP18 explained how these insights helped them to see a wider range of support options were likely to be appropriate for the patient, and how recording it in the patient’s notes would help provide context for future consultations:

*‘He came with quite specific ideas about what he wanted in terms of medication, but the outcome was that yes, medication seemed appropriate, but let’s also look at whether you could get support from the social prescribing team, whether there’s a role for social care as well […]. If I hadn’t done the training and he’d come in and said ”I’ve had a relapse of my depression I want to go back on the sertraline”, we’d have had a discussion, but I’d have been like “okay then, here you go, let’s see you in 4weeks”.’* (GP18)

The GP also felt that this discussion had provided opportunity for the patient to feel heard and to recognise the relevance of their circumstances on their mental wellbeing:

*‘I think [he felt] quite pleased that someone was showing an interest. And I don’t think he’d really thought of the relevance of his social circumstances to the anxiety and depression that he was experiencing.’* (GP18)

### Practice team working

GPs explained how better understanding of patients’ circumstances helped them to recognise and appreciate the role of the social prescriber and/or link workers in the practice team, including practice-based links to external agencies like the Citizens Advice Bureau, with several explaining they had increased referrals once they better understood the role of socioeconomic circumstances in patient distress:

*‘When the initial role of social prescriber came, I was very wary and […] we felt we will not use them […] But it was interesting that how, especially when we talk about poverty- related issues or mental health, I do feel they do play important role and we must involve them as a part of our team*.’ (GPNT02)

Increasing referrals were confirmed in interviews with other practice staff who felt their role was better understood following the training. Staff in supporting roles also explained how the training gave them the confidence to ask patients questions which had in turn helped relieve pressure on GPs:

*‘Before it would have been a case of whether they were on antidepressants or not – if they were telling me they’re depressed, I was telling them that they needed to see a GP. Now I’m emboldened to ask questions and try and identify what the issue is. Okay, if it’s to do with poverty then let’s try signposting and the social prescribers. […] And yeah, I’m getting a better response – more than just trotting out the old ‘we need to book and see a GP’ line*.’ (Healthcare assistant).

*‘Before they’ve come in and said, “I’m feeling this way”, I’d then straight away have to go and get a GP […] Now I feel so much more confident in saying “right, this is what we’ll do*”.’ (Practice nurse)

## Discussion

### Summary

Asking patients about socioeconomic circumstances was not common practice within primary care consultations. Lack of confidence, fear the patient may feel stigmatised, and uncertainty over how to respond to problems identified were key reasons for this. Following the DeStress-II training, health professionals recognised the value of asking such questions to better understand factors underlying patient distress. This in turn impacted on treatment, support and referral options, and better recognition of opportunities available through more multidisciplinary practice teams.

### Strengths and limitations

A study strength was the involvement of a wide range of GP practices covering diverse populations across England. The majority were based in areas of high deprivation where understanding socioeconomic circumstances is important to delivery of effective primary care. However, the DeStress-II training was also well received in more affluent areas with pockets of poverty, suggesting the core messages have wide application and may in fact be especially helpful for health professionals who do not confront poverty-related distress in everyday practice.

It is possible that practices already aligned with the approach advocated by DeStress-II were more likely to put themselves forward for the training than those who were not. However, interviews found that, for many, the training offered a new approach. Similarly, while participation in interviews was open to anyone in the practice team who had undertaken the training, it is possible that some bias in the data may be present if those opting to take part were particularly aligned with its aims. However, as well as discussing their own experience, participants were asked to discuss the training with all those who had attended and to report back on this wider experience during the interview. While no significant divergences of opinion within teams was reported, some GP interviewees reported that they or their GP colleagues felt that they already worked in the ways recommended in the training.

Interviews were undertaken within 3 months of the training; longer term follow-up is needed to understand whether changes to consultations are sustained and to assess impact on practice culture and patient wellbeing.

### Comparison with existing literature

This work coheres with existing literature exploring the challenges that health professionals face initiating difficult conversations around topics that are considered potentially uncomfortable, embarrassing, or stigmatising. However, much of this work is based on the discomfort and dilemmas of being supportive while suggesting that a patient change unhealthy behaviours.^3^ In contrast, our work advocates how understanding a patient’s socioeconomic circumstances can inform appropriate treatment or support and may reduce the likelihood of inappropriate prescribing. While most work in this area focuses solely on GPs, including allied health professionals in the training identified the potential for question-asking to lead to better practice team working.

Research has explored the possibility of flagging GP records to enable better targeting of poverty-related interventions. However, this is far from straightforward. Geographical data and area-based deprivation scores used in the UK are too blunt to characterise individual patient circumstance, and the Office for National Statistics explicitly states that the Indices of Multiple Deprivation should not be used for this purpose.^18^ Research on the use of income data to flag patient records in Canada has also highlighted the difficulties some people experience estimating their income or conveying their fluctuating financial circumstances.^19^ Other research has examined the benefits of including questions around ‘difficult’ issues as part of a standard GP assessment; for example, on suicide and substance use. While we agree that the use of such standard questions can be helpful, our findings agree with others who assert that they need to be embedded within the context of a broader experience that patients perceive to be meaningful and empathic engagement, rather than simply a ‘tick box’ exercise.^20^

Research has stressed that emotional work that remains unrecognised and unresourced impacts negatively on GP wellbeing and burnout.^21^ Some interviewees raised this as a potential concern, although it was not something they reported that they had experienced. Indeed, as others have found^11^ cultivating a deeper relationship with patients through a biopsychosocial approach was stated by some interviewees as potentially protective against burnout, at least in part because better understanding of patient need could facilitate team-based working.

### Implications for research and practice

Not asking patients about their broader life circumstances can neglect key triggers of underlying health problems, lead to problematic assumptions and inappropriate treatment and, in turn, potentially damage trust and the potential for therapeutic alliance between health professional and patient. With current resource pressures meaning that patients may not consistently see the same health professional, the importance of asking questions to ensure appropriate treatment and support pathways from the start are heightened. Primary care teams in England are now multidisciplinary, offering important opportunities for intra-team support. Asking questions about socioeconomic circumstances holds important potential for ensuring that the right person or set of people in the practice can provide the most appropriate patient support.

## Data

The dataset relied on in this article is available from the corresponding author on reasonable request.
